# Rotational atherectomy ablation for an unexpandable stent under the guide of IVUS: A case report

**DOI:** 10.1097/MD.0000000000009978

**Published:** 2018-02-16

**Authors:** Daoyuan Si, Guohui Liu, Yaliang Tong, Yuquan He

**Affiliations:** Department of Cardiology, China–Japan Union Hospital of Jilin University, Jilin Provincial Engineering Laboratory for Endothelial Function and Genetic Diagnosis of Cardiovascular Disease, Jilin Provincial Cardiovascular Research Institute, Changchun, Jilin, China.

**Keywords:** calcified plaque, intravascular ultrasound, rotational atherectomy, unexpanded stent

## Abstract

**Rationale:**

Inadequate stent expansion due to rigid calcified may result in restenosis lesions, but the available options are limited.

**Patient concerns:**

We report a case via the trans-radial approach of the severely underexpanded freshly deployed stent due to heavily calcified plaques

**Diagnoses:**

Coronary angiography revealed that there was no adequate expansion of the freshly deployed stent.

**Interventions:**

Under the guide of intravascular ultrasound (IVUS), rotational atherectomy (RA) successfully ablated the stent layers and the protruding calcified plaque. Followed by balloon angioplasty, the ablated segment was scaffolded with another stent, well expanded and documented by IVUS.

**Outcomes:**

The patient was uneventful during the procedure and remained angina free at the point of one year of clinical follow-up.

**Lessons:**

This case indicated that RA via the trans-radial approach could be a useful remedy in the situation of under-expansion of implanted stents, and the debulking should be performed under IVUS-guidance.

## 1 Introduction

Coronary stent implantation in severely calcified lesions may result in stent under-expansion, despite high-pressure inflation, thus potentially leading to stent thrombosis and restenosis.^[1,2]^ High-speed rotational atherectomy (RA) has been previously reported to be used in that scenario, but few reports have demonstrated RA both debulked metal component of the pre-existing stent and calcified plaques.^[3,4]^ Here we report a case, severely underexpanded freshly deployed stent due to heavily calcified plaques, via the trans-radial approach of RA-facilitated percutaneous coronary intervention (PCI) with the guide of intravascular ultrasound (IVUS).

## 2 Ethics

The study was approved by the Ethics Committee of China–Japan Union Hospital of Jilin University, China. The patient provided the written informed consent for the report.

## 3 Case report

A 70-year-old man with exertional angina was referred to our hospital, with a history of hypertension and dyslipidemia. Physical exam and laboratory findings were unremarkable. The electrocardiogram showed complete left bundle branch block, and transthoracic echocardiogram revealed left anterior wall hypokinesis.

**Figure 1:**
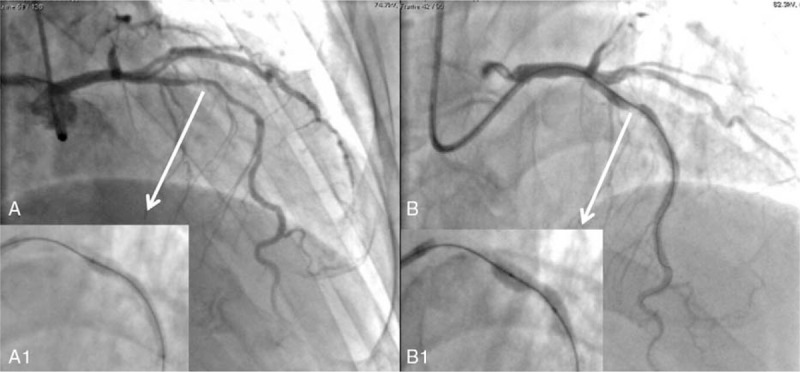
Angiography and undilated stent. (A) Baseline coronary angiogram of the LAD. (A1) The stent was deployed in proximal LAD (white arrow) with underexpansion. (B) Severe underexpansion of the implanted stent despite high-pressure inflation with the NC balloon. (B1) the NC balloon which cannot be fully expanded in stented proximal LAD (white arrow). LAD = left anterior descending artery, NC = noncomplaint.

**Figure 2:**
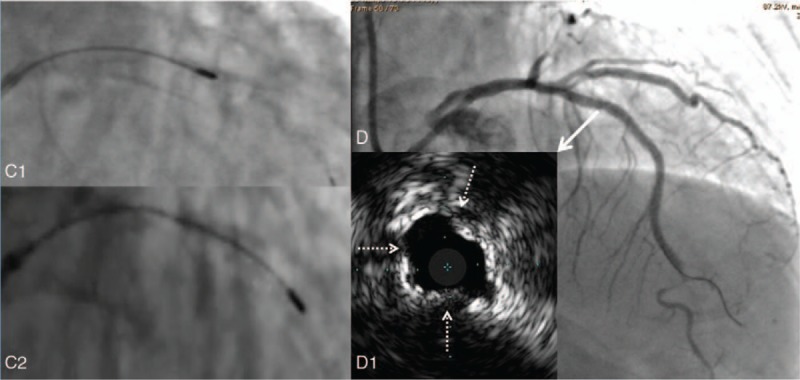
Rotational ablation of the stent C1, rotablator 1.25 mm burr passing through the stented proximal LAD. C2, rotablator 1.5 mm burr passed through the stented proximal LAD. D, after ablation and full expansion with a noncomplaint balloon, the angiography showed an acceptable result. D1, IVUS image confirmed ablation of stent implanted in proximal LAD (solid white arrow) at 1, 9, and 9 o’clock direction (white dotted arrow), but the minimum lumen area was only 5.6 mm^2^. LAD = left anterior descending artery, IUVS = intravascular ultrasound.

**Figure 3:**
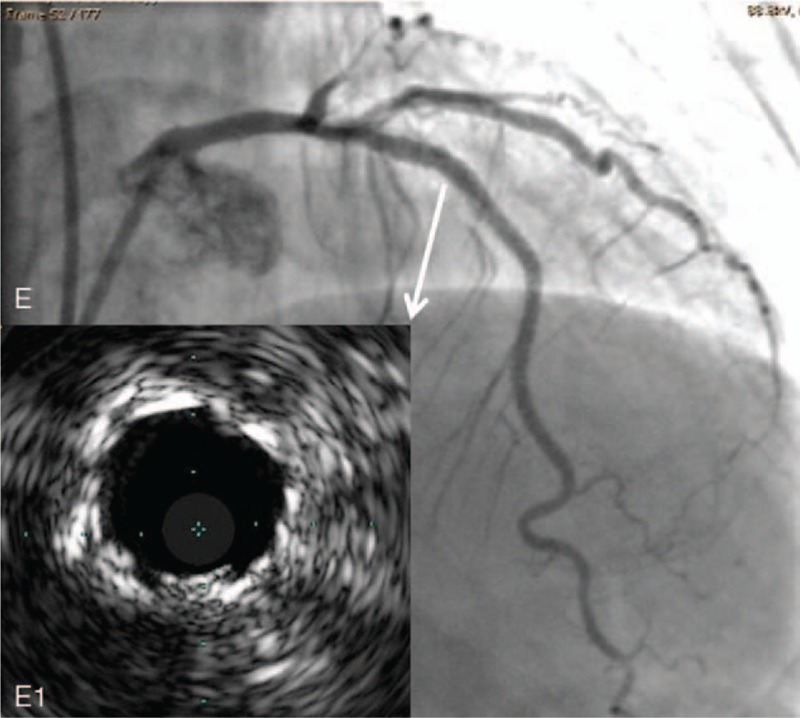
Final result. (E) The final angiogram showed fully expanded LAD stent. (E1) IVUS showed the bilayers stent in the proximal LAD (white arrow), and the final lumen area was 7.2 mm^2^. LAD = left anterior descending artery, IUVS = intravascular ultrasound.

Cardiac catheterization was then performed via the trans-radial approach. Coronary angiography revealed diffuse calcification from proximal to middle piece of the left anterior descending artery (LAD) with about 80% stenosis. The left circumflex artery (LCX) was tiny and right coronary artery had a moderately obstructive 50% stenosis with calcification. For the PCI of the LAD, a guidewire (Fielder XT, ASAHI) was used to cross the lesions. Then 12 atm dilation of a dual-wire balloon (ScoreFlex 2.0 × 15 mm, OrbusNeich) in the middle LAD was successful, but 14 atm dilation of the same balloon in the proximal LAD didn’t fully expand the calcified portion. Following this, a drug-eluting stent (DES) (Firebird 2.5 × 29 mm, Microport) was implanted in the middle LAD properly. With the parallel wiring technique, another DES (Partner 2.5 × 29 mm, Lepu) was implanted in the proximal LAD, but there was no adequate expansion of the focal calcified segment. Multiple inflations with noncompliant (NC) balloons (TREK 2.75 × 12 mm, Abbott; MONORAIL 2.75 × 12 mm, Boston), at pressures as high as 30 atm, failed to achieve sufficient post-dilatation (Fig. 1). IVUS catheter failed to pass through the stent, and RA (Rotablator, Boston) was then performed with the higher support of microcatheter (Finecross, Terumo). Under infusion of tirofiban, a 1.25 mm burr was gradually advanced to the lesion at 150,000 rpm and rotating speed always decreased unexpectedly at the proximal edge of the underexpanded stent. After multiple runs, the 1.25 mm burr finally passed through the lesion, but the noncompliant balloon (Sapphire 2.75 × 10 mm, OrbusNeich) still could not fully expand the stented lesion. Followed by a 1.5 mm burr passed at 150,000 rpm, the previous NC balloon at 20 atm achieved full expansion with a good angiographic result. Philips Volcano's IVUS interrogation of the stent segment confirmed that adequate ablation of the stent, but the 5.6 mm^2^ minimum lumen area was unacceptable (Fig. 2). Thus, another DES (Firebird 2.75 × 13 mm, Microport) was implanted with full expansion and followed with the dilation of an NC balloon at 28 atm (GRIP 3.0 × 8 mm, Acrostak). IVUS showed a satisfactory result with no stent malapposition or residual stenosis, and the final lumen area is 7.2 mm^2^ (Fig. 3). The patient was uneventful during the procedure and was discharged following a few days. At the point of one year of clinical follow-up, he remained angina free.

## 4 Discussion

Severely calcified lesions pose challenges to PCI because of high procedural risks and inadequate stent expansion which is strongly associated with restenosis and thrombosis.^[5]^ To avoid stent underexpansion, the stent should not be deployed in a nondilatable lesion in the first instance. Rotational atherectomy is often required to reduce plaque rigidity and to facilitate dilation before stent implantation, when aggressive predilatation fails to fully dilate the calcified lesion.^[6]^ But if you encounter the stent underexpansion due to rigid calcified lesions, the available options are limited when aggressive inflation of short noncompliant balloons can’t achieve full expansion.^[6,7]^ Rotational atherectomy has potentially shown to be a practical and effective method to duel with that intractable scenario in several case reports.^[8,9]^

However, the risk of entrapment and slow-flow are the major concern during RA being performed to ablate the stent.^[6]^ Compared to the calcified lesion, more rigid stent strut might make burrs more susceptible to entrapment. In our case, consistent with other reports,^[3,9]^ initially use of a small burr and a strategy of gradual, intermittent burr advancement may contribute to the prevention of the entrapment. A larger burr was used only after the noncompliant balloon couldn’t fully expand the stented lesion following the initially small burr. Slow flow during RA is considered to relate to microvascular embolization of plaque debris and associated thrombi.^[10]^ Metal particles produced by the stent ablation maybe not able to potentiate the microvascular embolization, because the metal particle was smaller than the diameter of an erythrocyte.^[11]^ In our case, the glycoprotein IIb/IIIa inhibitors were used following the recommendation and slow-flow did not arise.

Despite more than 10 cases of stent ablation was reported, few of them proved that the metallic component of the stent was removed by ablation of RA. In our case, IVUS revealed the disappearance of the stent strut caused by ablation. In addition, even though the angiographic result was acceptable after ablation and full expansion of the noncompliant balloon, IVUS image showed the lumen area of the stented lesion was unacceptable. This may enhance the concept that debulking should be performed under the IVUS guidance.^[12]^ Furthermore, to our knowledge, this is the first case reported using IVUS to guide the ablation for sirolimus-eluting stent via the trans-radial approach.

## 5 Conclusion

Although inadequate stents expansion due to rigid calcified is rare, the consequence is several including stent thrombosis and restenosis. This case indicated that RA could be a useful remedy in the situation of under-expansion of implanted stents, and the debulking should be performed under the IVUS guidance.
